# Evidence of Preferential Flow Activation in the Vadose Zone via Geophysical Monitoring

**DOI:** 10.3390/s21041358

**Published:** 2021-02-14

**Authors:** Lorenzo De Carlo, Kimberlie Perkins, Maria Clementina Caputo

**Affiliations:** 1 Water Research Institute, National Research Council of Italy, 70132 Bari, Italy; maria.caputo@ba.irsa.cnr.it; 2 U.S. Geological Survey, 345 Middlefield Rd., MS-420, Menlo Park, CA 94025, USA; kperkins@usgs.gov

**Keywords:** preferential flow, time-lapse ERT, moment analysis

## Abstract

Preferential pathways allow rapid and non-uniform water movement in the subsurface due to strong heterogeneity of texture, composition, and hydraulic properties. Understanding the importance of preferential pathways is crucial, because they have strong impact on flow and transport hydrodynamics in the unsaturated zone. Particularly, improving knowledge of the water dynamics is essential for estimating travel time through soil to quantify hazards for groundwater, assess aquifer recharge rates, improve agricultural water management, and prevent surface stormflow and flooding hazards. Small scale field heterogeneities cannot be always captured by the limited number of point scale measurements collected. In order to overcome these limitations, noninvasive geophysical techniques have been widely used in the last decade to predict hydrodynamic processes, due to their capability to spatialize hydrogeophysical properties with high resolution. In the test site located in Bari, Southern Italy, the geophysical approach, based on electrical resistivity tomography (ERT) monitoring, has been implemented to detect preferential pathways triggered by an artificial rainfall event. ERT-derived soil moisture estimations were obtained in order to quantitatively predict the water storage (m^3^m^−3^), water velocity (ms^−1^), and spread (m^2^) through preferential pathways by using spatial moments analysis.

## 1. Introduction

Preferential flow occurs in the vadose zone due to different hydraulic processes, often associated with obvious flowpaths such as biopores and fractures [1], but also in supposedly homogeneous media [2]. It usually moves water faster and with greater fluxes than other flow modes, driven by gravity with little influence of capillarity for a limited time in response to water input.

Preferential flow has implications for subsurface environmental processes, particularly on critical zone dynamics. The capability to identify preferential pathways in the subsurface is fundamental to determining the location and the mass of contaminants, to predict their migration and promptly prevent the spread in the hydrogeologic system. This is even more important considering that the critical zone plays a crucial role in several environmental questions. Specifically, the estimation of travel times of the infiltrated rainfall in the vadose zone can provide significant improvements in water storage management of aquifer recharge.

In agricultural water management, the water storage capability as a function of soil properties is no longer a trivial issue. In fact, in the last few years, precision agriculture strategy has become widespread based on the gathering, processing, and analysis of temporal and spatial data. The combination of such data with other information can be used to support management decisions according to estimated variability for improved resource use efficiency, productivity, quality, profitability, and sustainability of agricultural production. Furthermore, the water flow dynamics impact the water runoff with consequences for surface stormflow and flooding hazards.

Several mechanisms can trigger preferential flow patterns: (i) funneled flow [3] caused by heterogeneities of soil or rock hydraulic properties; (ii) fill-and-spill flow [4] along sloped and irregular subsurface layer; (iii) fingered preferential flow [5], related to heterogeneities of the moisture state and flux distribution; and (iv) macropore flow through elongated voids in soil and rock [6]. Understanding these hydrodynamics processes is critical for the quantification and prediction of PF caused by subsurface heterogeneity and the small scale at which flow processes take place.

Sensors typically used to measure the soil hydrological properties (time domain reflectrometry, time domain transmissometry or capacitive sensors) have only scarcely addressed preferential flow. An extended application of TDR for preferential flow properties in a controlled laboratory experiment was described in [7].

However, these sensors have limited applicability to detect preferential pathways at field-plot and larger scales for several reasons: (a) the small volume of sensitivity, (b) the invasiveness of the probes into the soil, and (c) the need to cover large areas with a great number of such sensors placed on the surface and at depth. Furthermore, typical sensors rely on the correlation to electric and dielectric properties of subsurface materials and, hence, provide an indirect measurement of the soil moisture.

Based on similar physical principles, noninvasive geophysical techniques have been widely used in the last decades to provide dynamic as well as static imaging of hydrological processes.

Among geophysical techniques, electrical resistivity tomography (ERT) and electromagnetic induction (EMI) are particularly suited to image fingered or funneled flow in the unsaturated zone due to the high sensitivity of the electrical resistivity to the subsurface soil moisture [8,9,10,11]. In several study cases, ERT was used to provide accurate estimations of soil moisture in hydrological applications [12,13,14,15,16,17,18]. Furthermore, ERT data have been assimilated with other multiple data sources, temperature and moisture content data, as well as hydraulic parameters to predict subsurface hydrological variables, typically hydraulic conductivity, and parameters [19,20,21,22,23].

In the test site of the Water Research Institute—National Research Council (IRSA-CNR), an artificial rainfall was induced in an experimental plot in order to monitor the activation of the preferential pathways in the soil. During the artificial rainfall event, time-lapse geophysical measurements have been collected to image the movement of the infiltrated water in the subsoil. The aim of the test was to see experimental evidence of preferential flow in the soil and to enhance knowledge about the mechanisms that trigger preferential pathways. The focus of this study was: (1) to recognize the capability of ERT to detect preferential pathways; (2) to provide quantitative estimation of the preferential flow in terms of mass balance and velocities; and (3) to highlight how the preferential patterns can change over time based on changes in water input, hence variation of the soil moisture distribution.

## 2. Materials and Methods

### 2.1. Study Site

**Figure 1:**
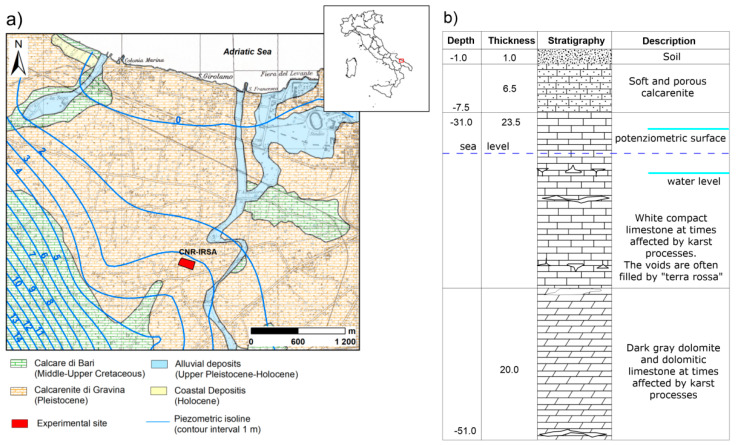
(**a**) Geological sketch of the investigated area; (**b**) stratigraphic column.

The experimental site is located within IRSA-CNR, Department of Bari (41°07′ N and 16°49′ E, 16 m a.s.l.), Southern Italy (Figure 1).

From a geological point of view, a thin layer of soil overlaps sedimentary carbonatic porous sandstone of marine origin belonging to Quaternary deposits. Below, a thick Mesozoic sedimentary succession, made up of limestone with frequent intercalations of dolomitic limestones and grey dolostones, constitutes the main aquifer and includes the lower part of vadose zone. Moreover, little canyons, locally named “lame”, formed by erosive activity of ephemeral streams, are filled with upper Pleistocene–Holocene deposits, made up of carbonate gravels with a reddish fine-grained matrix. In the study area, accurate stratigraphic details have been provided by five drilling wells widely used in the past years for hydrogeological tests. The central well with largest diameter is surrounded by four wells, drilled 10 m apart along the cardinal directions. The soil is a mixture of bare and waste soil, making core sampling difficult both for technical reasons and for the poor representativeness of soil properties of the larger studied area. The thickness of the soil is about 1 m on average. This information was used to plan the geophysical survey parameters, including resolution and depth of investigation.

### 2.2. Field Experimental Set Up: The Irrigation System

**Figure 2:**
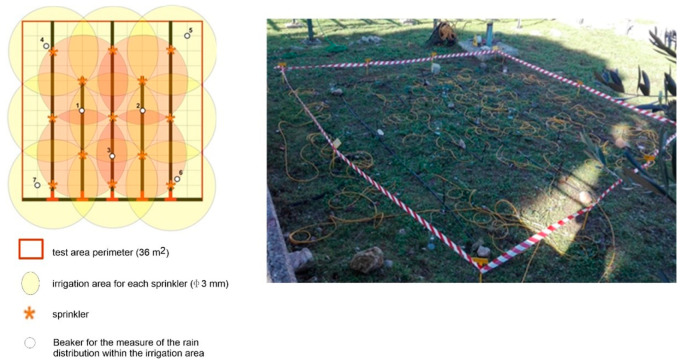
Experimental set up for the artificial rain test and for ERT monitoring.

Several irrigation cycles were performed between 5 December and 15 December 2018. The rainfall event was simulated by placing on the ground a network of 13 sprinklers located in an area of 36 m^2^ to provide a uniform distribution of water droplets in the whole experimental area (Figure 2). A pipe system connected to the sprinklers provided 2 m^3^ of water in five hours, corresponding to an artificial rainfall of about 7 mm h^−1^.

### 2.3. Electrical Resistivity Measurements and Inversion Problem

Noninvasive electrical resistivity measurements are particularly suited to monitoring the hydrodynamic processes of the subsurface due to the strict analogy between electrical and hydrological features. Particularly, the electrical resistivity of soil and rocks mainly depends on fluid flow into the medium through porous space and fractures, in the absence of significant soil temperature changes. Resistivity measurements are usually performed by using a four-electrode array, a pair of electrodes used to inject electrical current into the ground and another pair to measure the potential voltage.

According to the Ohm’s law, the electrical resistance is the ratio between the measured potential voltage and the injected electric current. The electrical resistivity, i.e., the inverse of the electrical conductivity, is proportional to the resistance based on a geometrical factor that depends on the arrangement of the four electrodes [24].

When the subsurface is assumed homogeneous, the observed electrical resistivity is apparent; however, due to common subsurface heterogeneities, deriving a true subsurface resistivity model requires solving the inversion problem [25].

The inversion of the data is a numerical procedure that, starting from an initial resistivity distribution, modifies the model iteratively so that the difference between the model response and the observed data values is minimized. Collecting the resistivity measurements in the time-lapse mode, i.e., repeating the measurements over time along the same profiles, the resistivity variations can be associated to changes of the hydrological condition of the investigated medium.

#### 2.3.1. Sensitivity Analysis

The sensitivity analysis is a useful tool to obtain an estimate of the reliability of the planned ERT survey in terms of design array configuration and data acquisition strategies.

The spatial sensitivity distribution is a property of the array configuration and does not change with the subsurface properties. The sensitivity map shows, given the selected electrode array, how much a change in resistivity in a block of the model affects the potential readings. The higher the sensitivity, the more reliable the final resistivity value of the block. Mathematically, the sensitivity function (known also as Jacobian matrix) represents the relationship that links the variation of the apparent resistivity data to a change in the subsurface resistivity [26]. For each quadrupole, the analytical solution of the Jacobian matrix was calculated for a homogeneous medium. As rule of thumb, blocks with sensitivities above a 0.01 threshold (1% of the maximum) have to be considered as significantly influenced by the measurements [27].

In the sensitivity domain, a grid of 48 electrodes, eight along *x* axis 0.7 m apart and six along *y* axis 0.9 m apart, the same grid used at field plot-scale, was set to detect geophysical features of the investigated soil volume. A combination of 1035 dipole–dipole resistance measurements with skip 0 (dipoles with minimal distance equal to one electrode spacing) was used to generate the sensitivity map.

#### 2.3.2. ERT Monitoring

The time-lapse resistivity measurements were collected for three days, from 5 December to 8 December 2018. Background resistivity was recorded before the starting of the test; then, more than 80 independent ERT datasets were collected every hour, each of them made of 1035 dipole–dipole resistivity measurements, the same data sequence generated in the sensitivity analysis.

The resistivity meter Syscal Pro Switch 48 (Iris Instruments, Orleans, France) was used to collect field data and, in order to respect the data frequency collection during the whole monitoring period, ComSys code was used to manage the ERT acquisitions in remote mode. Each acquisition took about 13 min. Before initiation of each data collection, the resistance contact between electrodes and soil was recorded to ensure a good flow of the electrical current into the ground. Transmission parameters (injection pulse duration 250 ms, number of stacks for each measurement between 3 and 6, and the maximum standard deviation of the measurements in a cycle of 5%) were adequately set to collect good quality data.

The first step of the inversion procedure consisted in removing from the whole ERT dataset the outliers that exceed threshold values of injected current (*I* < 1 mA) and standard deviation between measurement cycle stacks of Q > 5%.

From the overall ERT data set, we removed about 4% of the raw data; hence, for each dataset, the total number of measurements considered for the inversion was 994.

An approach based on the inversion of the resistance ratios measured at different time points with respect to the background was used to enhance the changes in electrical resistivity over time. This approach tracks small resistivity changes over time and minimizes the modeling errors and data errors due to imaging artifacts that typically would occur in a standard inversion.

Moreover, compared to the ratio of the inverted independent ERT images, the inversion of the resistivity ratio is a more refined and robust procedure to highlight the water movement in the vadose zone [28].

For each quadrupole, the recalculated resistance was:

(1) $$R=\frac{R_{t}}{R_{0}}R_{\mathrm{ohm}},$$

where *R_t_* is the resistance measured at *t*, time, *R*_0_ is the background resistance measured before starting the infiltrometer test, and *R*_ohm_ is the resistance for a homogeneous medium chosen to have a uniform resistivity equal to 100 Ωm, calculated by averaging the whole set of apparent resistivity ratios.

The full three-dimensional inversion of apparent resistivity was performed using ERTLab3D inversion software, developed by Multi-Phase Technologies (Sparks, NV, USA) and Geostudi Astier (Livorno, Italy), based on a smoothness-constrained inversion scheme, which uses a Finite Elements (FEM) approach to model the subsoil.

For each ERT dataset, the initial model was chosen as the median value of the apparent resistivity measured in the field and was iteratively modified to minimize an objective function, based on the difference between the model response and the observed data values. A robust inversion, based on data variance iterative reweighting, was used to appropriately manage the effect of non-Gaussian noise [29]. The ERTLab code uses a tetrahedral structured finite element mesh, settled by the user equal to the half of the inter-electrode spacing in all directions (0.35 m along *x*-direction, 0.48 m along *y*-direction, 0.35 m along *z*-direction) for a total numbers of nodes of the mesh equal to 41,667 nodes and 37,800 blocks. The convergence was reached after 5–6 iterations.

#### 2.3.3. ERT-Derived Soil Saturation Degree

According to the procedure adopted in [30], a formulation of Archie’s law [31] was used to convert the inverted resistivity ratios directly into saturation degree

(2) $$\rho \left(t_{i}\right)=\rho \left(t_{0}\right){\left(\frac{S_{w}\left(t_{0}\right)}{S_{w}\left(t_{i}\right)}\right)}^{\;n},$$

where ρ(*t*_0_) and ρ(*t*_1_) are the resistivity values of the soil (Ωm) at times *t*_0_ and *t*_1_, respectively, and S_w_ (*t*_0_) and S_w_ (*t*_1_) are the saturation degrees (volume of water to the volume of voids) at times *t*_0_ and *t*_1_, respectively. This approach allows the simplification of Archie’s equation, because the “*n*” saturation index is the only unknown parameter to be calibrated in the laboratory. The n saturation index was calibrated in the laboratory and set equal to 2. Thus, the saturation degree or soil moisture ratio can be directly estimated from the inverted resistivity ratio. The background soil moisture was set to 0.20 m^3^m^−3^, the value measured before the starting of irrigation by sensors installed in the gauge station, located 5 m from the experimental area. Temperature sensors installed into the soil recorded no significant temperature changes during the whole monitoring period.

### 2.4. Moment Analysis

The spatial moments were inferred from ERT data to provide a quantitative estimation of the preferential flow dynamics in terms of change in water storage, water velocity, and vertical spread of the mass triggered by a vertical infiltration. Several study cases concerning the moment analysis for hydrological and geophysical applications have been already published [32,33,34].

The basics of the moment analysis is the following equation:

(3) $$M_{ijk}\left(t\right)={∭}_{\Gamma }\Delta \theta \left(x,y,z,t\right)x^{i}y^{j}z^{k}dxdydz.$$

The zeroth, first, and second spatial moments correspond to *i* + *j* + *k* = 0, 1, and 2, respectively.

Δθ is the water content changes based on the resistivity changes estimation inferred from the time-lapse ERT model, after removing the background water content. Γ is the volume of interest.

In 2D dimensions, the zeroth moment, *M*_00_, is the changes in water mass within the domain respect to the background and represents the water storage, expressed in m^3^ m^−3^.

(4) $$M_{00}\left(t\right)=\iint \Delta \theta \left(x,z\right)dxdz.$$

The first moment, *M*_01_ normalized by the mass *M*_00_, defines the vertical center of mass of the plume at a given time, *z* (Equation (5)).

(5) $$z=\frac{M_{01}}{M_{00}}.$$

The vertical spread of the mass about its center, *σ* (m), is related to the second spatial moment, *M*_02_.

(6) $$\sigma_{zz}^{2}=\frac{M_{02}}{M_{00}}-{\left(\frac{M_{01}}{M_{00}}\right)}^{2}.$$

## 3. Results

### 3.1. Sensitivity Model

**Figure 3:**
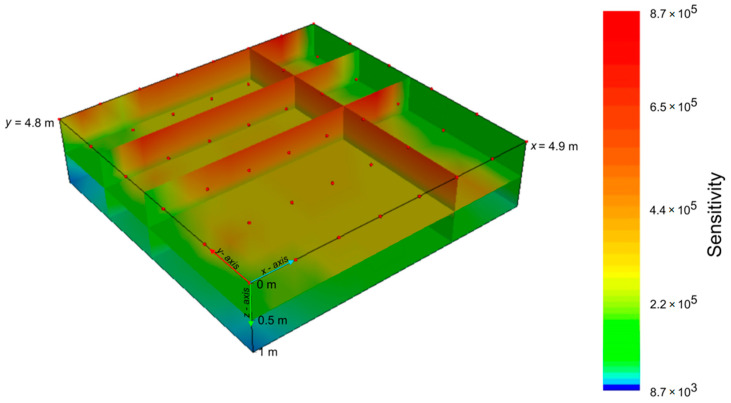
Sensitivity analysis performed on ERT data.

Figure 3 shows the 3D sensitivity analysis of the global set of measurements for a homogeneous resistivity model. The value for each block is the sum of the individual quadrupole sensitivities. The lower limit of the sensitivity map has been set equal to the 1% with respect to the higher one, in order to evaluate the portion of the investigated domain mainly influenced by the resistivity measurements.

Excepting some parts of the lower boundaries of the domain, the map shows high values of the sensitivity and confirms the effectiveness of the adopted experimental scheme in providing an accurate ERT soil model.

### 3.2. ERT Monitoring Results

**Figure 4:**
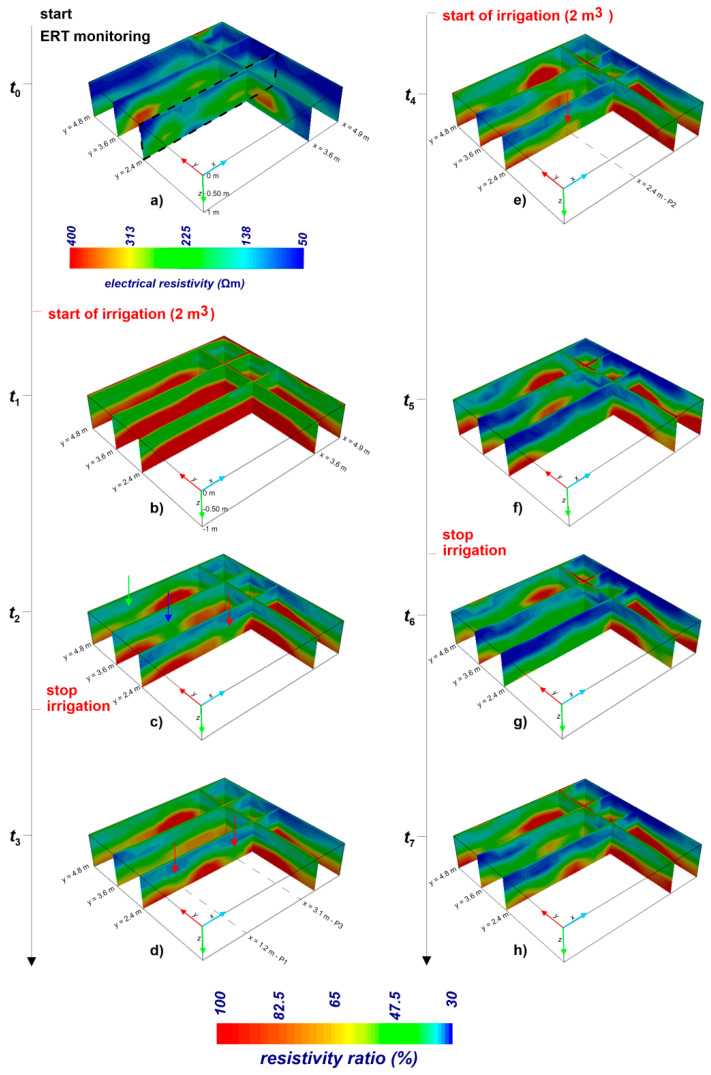
Time-lapse ERT results. Times from *t*_0_ to *t*_7_ are related to: (**a**) ERT before irrigation (reference time *t*_0_); (**b**) 54 min from *t*_0_; (**c**) 257 min from *t*_0_; (**d**) 643 min from *t*_0_; (**e**) 1243 min from *t*_0_; (**f**) 1453 min from *t*_0_; (**g**) 1543 min from *t*_0_; (**h**) 2113 min from *t*_0_.

Figure 4b–h shows the most significant inverted ERT ratio images collected during the whole monitoring period.

The background resistivity, i.e., the resistivity distribution at reference time *t*_0_, visualizes a snapshot of the soil condition before the water injection (Figure 4a). The static resistivity varies from 50 to 400 Ωm, although the main electrical signal is within 250 Ωm. In terms of textural properties of the soil, the narrow resistivity range can be interpreted as heterogeneities due to the presence of debris material or different compaction degrees. The resistivity changes shown in the time-lapse analysis are attributed to the hydrological variations caused by the water infiltration during the irrigation test. The color scale was between resistivity ratio of 30 and 100; 100 means that no resistivity changes are observed with respect to the background conditions; 30 means that highest changes are observed due to the water infiltration.

At time *t*_1_, corresponding to 54 min from *t*_0_ (Figure 4b), a clear surface discontinuity is observed over the whole investigated volume at about 0.50 m below ground surface (bgs). Above this surface, the observed resistivity is decreased to about half the background condition, while below this surface, no changes are recorded. In terms of hydrological features, the water infiltrates homogeneously in the upper part of the soil, i.e., above the discontinuity surface, with a significant decrease (about 50%) of the resistivity distribution.

At time *t*_2_, 257 min from *t*_0_ (Figure 4c), the discontinuity surface is interrupted in several parts of the investigated volume (red arrow in the section *y* = 2.4 m, blue arrow in the section *y* = 3.6 m, and green arrow the section *y* = 4.8 m), showing clear evidence of preferential flow, probably funneled and fingered flow mode together. The strong heterogeneity in preferential pathways over very small spatial domain is confirmed at time *t*_3_, 643 min from *t*_0_ (Figure 4d), where other patterns are triggered in some portions of the domain. Particularly, we focus our attention on the reference section *y* = 2.4 m, which shows how the preferential water movement is triggered over time at very small spatial scale, depending on soil heterogeneity and on soil moisture state. In fact, in addition to the preferential flow observed at time *t*_1_ (red arrow in point P3), another preferential pattern activates in point P1.

Moreover, after the start of the second irrigation cycle (time *t*_4_, 1243 min from *t*_0_ and time *t*_5_, 1453 min from *t*_0_, shown in Figure 4e,f), the time-lapse geophysical monitoring highlights two main effects: (1) above the surface discontinuity, the significant decreasing of the electrical resistivity, as depicted by the deep blue color, represents an increase in soil moisture; (2) below the discontinuity, a decrease in resistivity is recorded in correspondence to the resistive body at the point P2 (red arrow in the picture), due to the infiltration of water in that portion of the domain. This trend is observed up to time *t*_6_, 1543 min from *t*_0_ (Figure 4e–g), causing an attenuation of the preferential flow effect. At time *t*_7_, 2113 min from *t*_0_, in correspondence to the point P2, an increase in resistivity due to the drying effect is observed in the deep layer of the domain.

**Figure 5:**
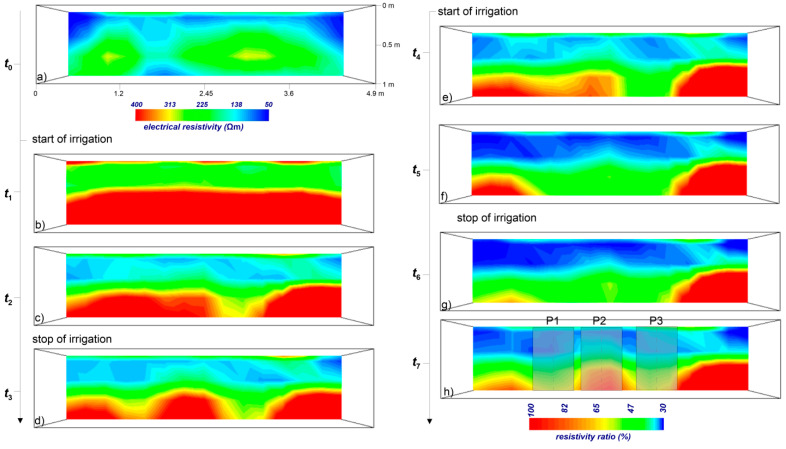
2D ERT sections extracted at *y* = 2.4 m at the same time points shown in Figure 4. Times from *t*_0_ to *t*_7_ are related to: (**a**) ERT before irrigation (reference time *t*_0_); (**b**) 54 min from *t*_0_; (**c**) 257 min from *t*_0_; (**d**) 643 min from *t*_0_; (**e**) 1243 min from *t*_0_; (**f**) 1453 min from *t*_0_; (**g**) 1543 min from *t*_0_; (**h**) 2113 min from *t*_0_.

This evidence supports the hypothesis of the strong spatial and temporal variability of the flow dynamics in the vadose zone, strictly related to the hydrological properties of the soil and to the water input. The trend of funneled and/or fingered flows described above is shown in Figure 5 where 2D pictures were extracted from 3D resistivity volume along section *y* = 2.4 m. The distance between points P1 to P3 is less than 2 m, pointing out that preferential flow could show unexpected behavior at a very small spatial domain.

### 3.3. ERT Derived Soil Moisture Profiles

In order to provide a hydrological meaning to the geophysical images, ERT derived soil moisture vertical profiles have been obtained by using the simplified Archie’s law described in (3). Particularly, setting the average porosity of the soil *φ* = 0.4 from laboratory measurements, the soil moisture profiles were inferred in correspondence to the three specific points P1, P2, and P3, respectively, shown in Figure 5h. These points are related to the locations where significant resistivity changes were observed over time, based on the ERT images. Blue lines are related to the first irrigation cycle, red ones to the second irrigation cycle.

**Figure 6:**
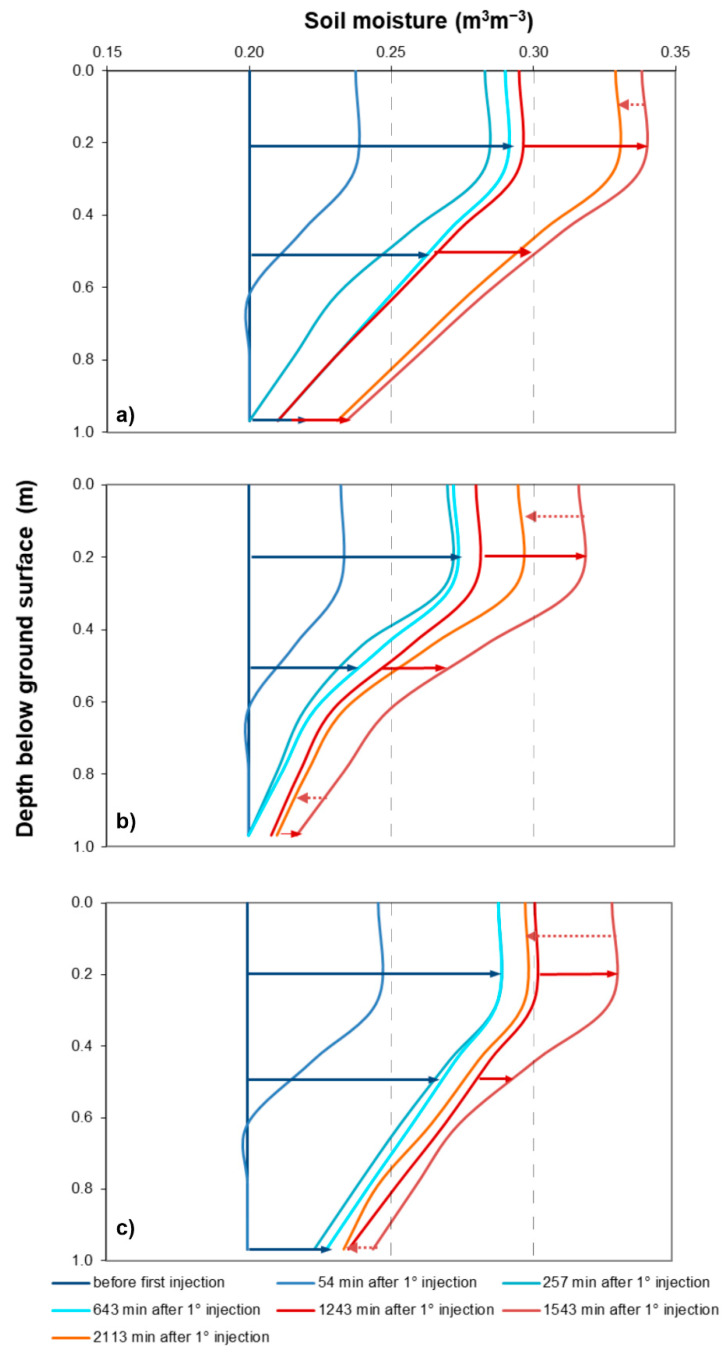
ERT-derived soil moisture profiles in: (**a**) point P1; (**b**) point P2; (**c**) point P3.

The soil moisture increases after the start of each irrigation cycle. The increase is steady but not uniform for the three observation points. Continuous blue and red arrows show the magnitude of the soil moisture increase at 0.20, 0.5, and 1 m bgs, after the first and the second irrigation cycles, respectively, while magenta dotted arrow highlights the decrease after the stop of the second irrigation cycle, corresponding to the end of the geophysical monitoring (Figure 6).

The graphs clearly demonstrate spatial and temporal variations in water infiltration caused by soil saturation conditions and the water input. Comparing the two irrigation effects, the increase in soil moisture is much higher after the first irrigation, due to initial low soil moisture conditions. The main differences between the three observation points are recorded below 0.5 m from ground surface. At time *t*_2_, 54 min from *t*_0_, a marked increase in soil moisture is observed in P3 (θ = 0.27 m^3^m^−3^) at 0.5 m bgs, while in P1 and P2 the soil is drier (θ = 0.25 m^3^m^−3^). This trend is even more enhanced at 1 m in depth. In fact, at time *t*_3_, cyan line corresponding to 643 min after time *t*_0_, the water is infiltrating in P1 (θ = 0.23 m^3^m^−3^) and in P3 (θ = 0.24 m^3^m^−3^), while no infiltration is recorded in P2 (θ = 0.2 m^3^m^−3^). After the start of the second irrigation cycle, the water also infiltrates in the deep layers in correspondence to the point P2 (θ = 0.21–0.22 m^3^m^−3^ at 1 m bgs), with more distributed water in the overall domain, which causes the preferential flow to stop.

At the end of the monitoring (time *t*_7_, 2113 min from *t*_0_), a decrease in soil moisture, due to the drying effect, is observed in correspondence with the stop of the second irrigation cycle. Similarly, the soil desaturates in different ways, with more marked changes in soil moisture in P2 and P3. Particularly, in point P2, the soil moisture reaches minimum values (θ = 0.21 m^3^m^−3^ at 1 m bgs) comparable with those observed before the irrigation test, highlighting a rapid desaturation process.

This peculiarity confirms the trend observed in the ERT images (Figure 4h and Figure 5h), highlighting the capability of the presented approach to capture details in preferential flow activation/break and in the rate of the flow dynamics.

### 3.4. Moment Analysis Results

**Figure 7:**
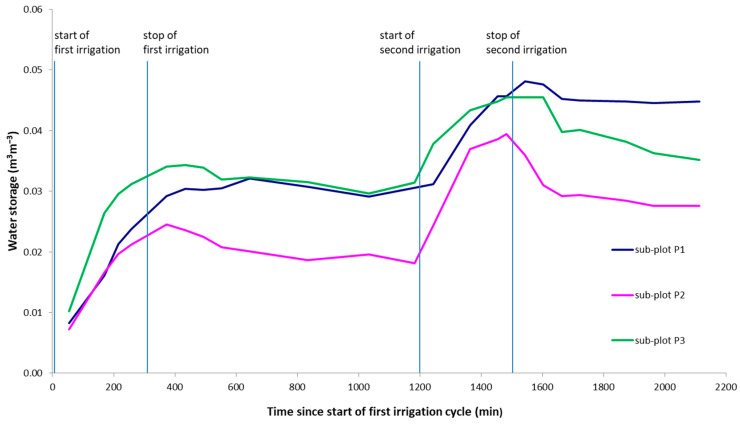
Water storage estimated in the three sub-plots through zeroth moment *M*_00_.

**Figure 8:**
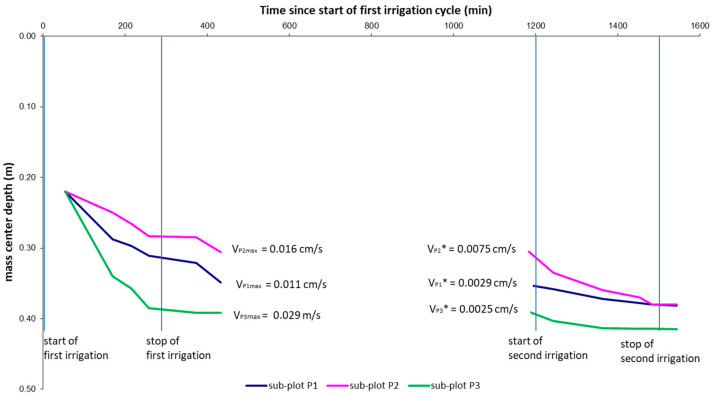
Depth of the mass center reached in the three sub-plots after two irrigation cycles.

**Figure 9:**
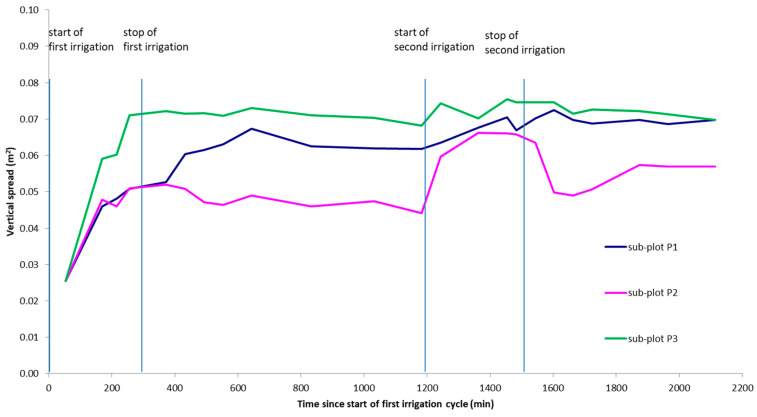
Vertical spread for the three sub-plots obtained in the whole ERT monitoring period.

2D spatial moment analysis was calculated along three sub-plots of the section *y* = 2.4 m centered in points P1, P2, and P3, where different preferential pathways were observed at very small distances. The water storage, the location of the mass center, and vertical spread of infiltrated water inferred from time-lapse ERT models were estimated using Equations (4)–(6), respectively, as plotted in Figure 7, Figure 8 and Figure 9.

Figure 7 highlights the different estimated water storage that moves in the three sub-plots. In correspondence of the start of the two irrigation cycles, the increase in soil moisture, which leads to an increase of water storage, can be seen. The slight delay time of the peak observed in sub-plot P1 can be related to the temporal resolution of the ERT acquisitions. In sub-plot P3, the water storage, corresponding to the area below the curve, is significantly higher compared with that observed in sub-plots 2 and 3, indicating that the water infiltrated differently, despite the small distance between the sub-plots analyzed.

Figure 8 shows the center of mass vertical motion, computed from ERT images.

After the first injection, the slope of the three lines highlights how the mass center of the infiltrated water moved downward with different velocities.

In fact, along sub-plot P3 (green line corresponding to the location *x* = 3.1), after the stop of the first irrigation, i.e., 433 min from *t*_0_, the mass center deepens up to 0.40 m from bgs much more than for sub-plot P1 (0.31 m from bgs) and sub-plot P2 (0.28 m from bgs). In sub-plot P3, the estimated velocity is about 0.029 ms^−1^, twice with respect to the sub-plot P1 and more than twice with respect to the sub-plot P2.

The second irrigation cycle highlights the water movement dependence from the starting soil water content conditions. In fact, the depth of the mass center deepens much more in sub-plot P2, where the soil moisture was lower than in sub-plots P1 and P3. Consequently, the infiltration rate in correspondence to the sub-plot P2 is three times higher compared with those estimated in sub-plots P1 and P3.

The vertical spread of injected water is plotted in Figure 9. The graph shows an increase in vertical spread over the experiment as water migrates downward. After the first injection, the spread is significantly higher in sub-plot P3 compared with the sub-plots P1 and P2, while the second injection causes an increase of the spread in sub-plot P2, confirming the trend of the first and second moment.

## 4. Discussion and Conclusions

The comprehension of water flow dynamics requires a basic support of innovative, feasible, and low-cost approaches. This is even more important when the flow processes lead to preferential patterns due to the great heterogeneity of the vadose flow and nonlinearities of their properties and processes. In addition, the variable hydrological properties at the soil–rock interface, the moisture conditions changing over time, and the character of the weather events contribute to the complexity of such phenomena.

The preferential flow can be funneled, caused by heterogeneities of soil or rock hydraulic properties, and fingered, related to heterogeneities of the moisture state and flux distribution, macropore flow, or a combination of such modes. Preferential flow phenomena are fast, unpredictable and strongly variable over time and, for this reason, they are difficult to study. In this context, the geophysical measurements have proved to be a promising tool for the monitoring of the hydrological processes, where point scale sensors (TDR, TDT, capacitance sensors, etc.) typically used for several applications are inadequate to capture the strong field scale heterogeneities. Unlike any other method, geophysics are able to image the subsurface directly, and they can help to identify the best locations for direct monitoring equipment if resources are limited. Their capability to spatialize physical properties, strictly related to the hydrological variables, can fill in the knowledge gap and overcome the lack of detailed information on the processes that occur in the vadose zone.

In fact, the geophysical tool can provide answers to the main problems involving preferential flow patterns. It can be helpful to identify where preferential pathways are triggered, how they relate with soil moisture conditions, how the flow spreads, and how fast these dynamics evolve. This information could have relevant practical implications on the prevention and prediction of the impact of natural or human-induced events, where a clear understanding of preferential flow processes and accurate input data are necessary to build robust and reliable models. In light of these considerations, this approach can be used as a powerful resource in the service of the main environmental issues, such as risk prevention and water resource management.

In fact, damage and disaster risks caused by oil spills, contaminant spread, and infiltration of toxics from the surface, which dangerously propagate in the vadose zone towards the aquifer, require detailed knowledge of these dynamics. At the same time, in the context of climate change mitigation and adaptation, several actions adopted in stressed or water scarce conditions, such as the use of unconventional wastewater in agriculture or water storage in the managed aquifer recharge systems, need real-time monitoring of the flow dynamic in order to evaluate the impact of these actions on the critical zone ecosystems.

In this study, during an experiment of artificial rainfall, the infiltration dynamics within the vadose zone were observed through time-lapse geophysical survey. The geophysical analysis produced evidence of preferential flow within very small scale in the spatial domain. Evidence of these patterns descend from different data processing, both qualitatively by visualizing changes in geophysical images over space and time, and quantitatively, from vertical soil moisture profiles and moment analysis, although deriving from a non-direct approach.

Particularly, the capability for predicting water storage and water velocity with high resolution in the 4D space–time dimensions was effective to enhance knowledge about the mechanisms that trigger preferential pathways. In this regard, the soil moisture distribution plays a crucial role for activation of the flow patterns rather than water flow rate that infiltrates into the soil. In fact, by considering that the two irrigation cycles had the same flow rate (2 m^3^) and assuming a homogeneous background soil moisture condition, the hydrological response shows a clear dependence on the soil moisture variations over time. Preferential flow is triggered in some points of the domain faster and more intensely, while in other points, it is activated when a threshold of soil moisture is overcome and preferential flow stops rapidly when the soil moisture decreases.

The results of the proposed study point out that time-lapse geophysical ERT surveys can be routinely adopted as a powerful investigative tool for a better comprehension of the flow and transport processes in the vadose zone. It is a low cost and effective solution to track preferential flow over large areas better than the conventional measurement systems currently used, by providing qualitative imaging of the flow dynamic and quantitative estimations of the variable water storage, velocity, and spread.

## Data Availability

Not applicable.
